# Supplementary material for: Macrophage deletion of Noc4l triggers endosomal TLR4/TRIF signal and leads to insulin resistance
Source: Nat Commun. 2021 Oct 21;12:6121. doi: 10.1038/s41467-021-26408-3 (PMC8531303; doi:10.1038/s41467-021-26408-3)
Supplement: Supplementary file 1 — Supplementary Information [file 41467_2021_26408_MOESM1_ESM.pdf]

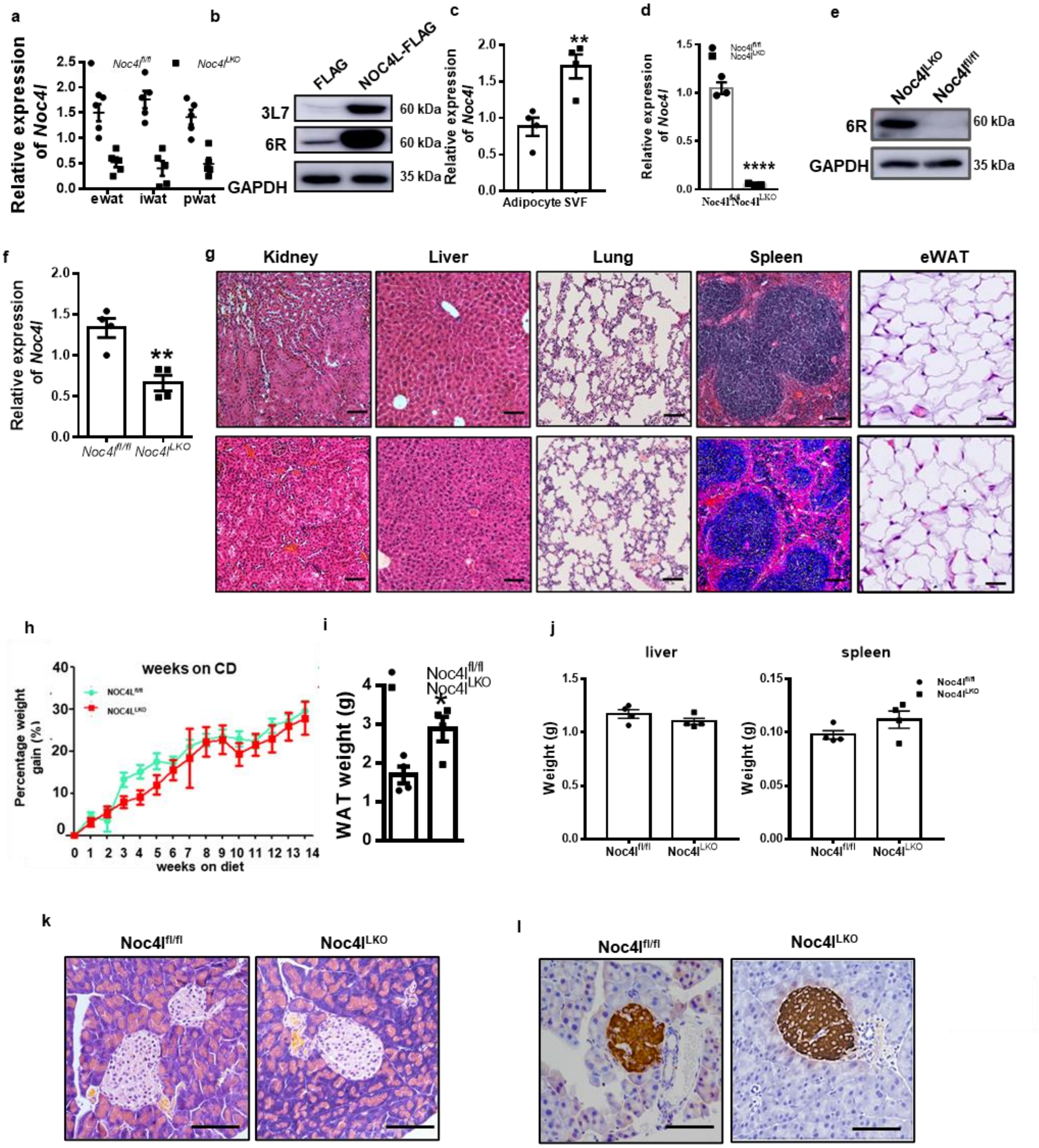

a
Relative expression of Noc4l
Noc4l^fl/fl
Noc4l^LKO
ewat
iwat
pwat
b
FLAG
NOC4L-FLAG
3L7
6R
GAPDH
60 kDa
60 kDa
35 kDa
c
Relative expression of Noc4l
Adipocyte SVF
**
d
Relative expression of Noc4l
****
e
Noc4l^LKO
Noc4l^fl/fl
6R
GAPDH
60 kDa
35 kDa
f
Relative expression of Noc4l
**
Noc4l^fl/fl
Noc4l^LKO
g
Kidney
Liver
Lung
Spleen
eWAT
h
weeks on CD
Percentage weight gain (%)
NOC4L^fl/fl
NOC4L^LKO
weeks on diet
i
WAT weight (g)
Noc4l^fl/fl
Noc4l^LKO
*
j
liver
spleen
Weight (g)
Noc4l^fl/fl
Noc4l^LKO
k
Noc4l^fl/fl
Noc4l^LKO
l
Noc4l^fl/fl
Noc4l^LKO

Supplementary Fig. 1 Phenotype of Noc4l ^{LKO} mice.

(a) The mRNA expression levels of *Noc4l* in eWAT, iWAT and pWAT from WT and Noc4l^{LKO} mice (n = 5 per group). (b) Protein expression of NOC4L extracted from NOC4L-FLAG overexpressed HEK293T cells using the prepared mouse monoclonal antibody (3L7) and rabbit polyclonal antibody (6R) (n = 3 independent experiments). (c) Relative mRNA expression of *Noc4l* in the adipocyte and stromal vascular fractions (SVF) isolated from the eWAT of C57/BL6 mice (n=4). **p =0.0093. (d-e) Noc4l expression in BMDMs from Noc4l$^{fl/fl}$ and Noc4l^{LKO} mice as assessed by RT-qPCR (n = 3 biological replicates) (d) and western blotting (e). ****p <0.0001 (d). Data represented three independent experiments (e). (f) Relative expression of Noc4l in ATMs from Noc4l$^{fl/fl}$ and Noc4l^{LKO} mice (n = 4 per group). **p =0.0043. (g) Histological analysis of kidney, liver, lung, spleen and eWAT of Noc4l$^{fl/fl}$ and Noc4l^{LKO} mice by H&E staining. Data represented three independent experiments. Scale bar, 50 μm. (h) Weight gain percentages of Noc4l$^{fl/fl}$ (n = 12) and Noc4l^{LKO} (n = 7) mice fed with CD for 14 weeks. (i-j) The mass of eWAT , liver and spleen in Noc4l$^{fl/fl}$ and Noc4l^{LKO} mice on HFD for 20 weeks (n=4 per group). *p =0.0216 (i). (k-l) Histological analysis and immunochemistry (insulin) of pancreas from Noc4l$^{fl/fl}$ and Noc4l^{LKO} mice. Data represented three independent experiments. Scale bar, 50 μm. Two-tailed Student's t test (c, d, f and i). All data are presented as mean ± SEM. *p < 0.05, **p < 0.01, ***p < 0.001.

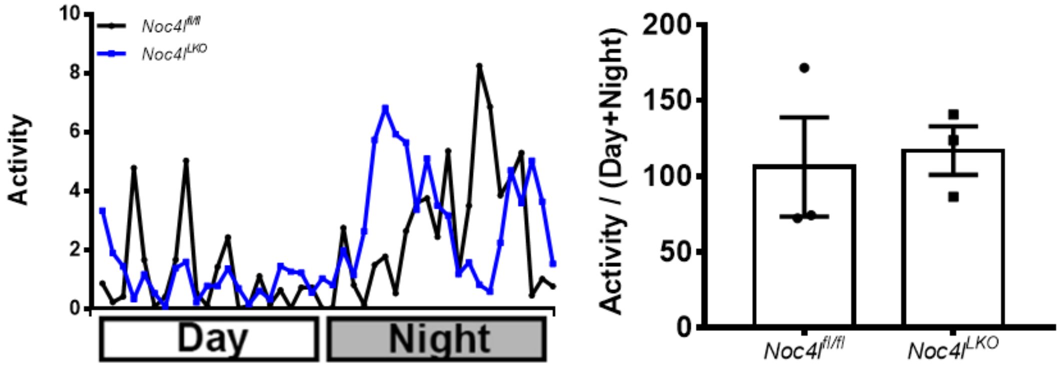

Supplementary Fig. 2 The activity of Noc4l$^{fl/fl}$ and Noc4l^{LKO} mice.

The locomotor activity of Noc4l$^{fl/fl}$ and Noc4l^{LKO} mice (n=3 per group). Quantification of the activity was shown in the right panel. All data are presented as mean ± SEM.

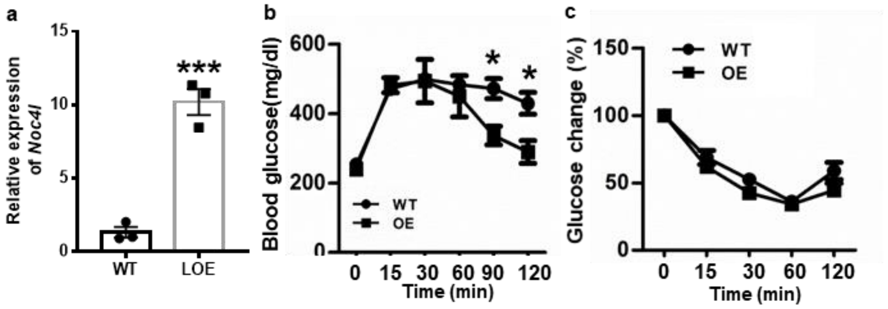

Supplementary Fig. 3 The GTT and ITT of WT and Noc4l^{LOE} mice.

(a) The expression of Noc4l in BMDMs from WT mice and Noc4l^{LOE} mice (n = 3 per group). Two-tailed Student's t test. ***p =0.0007. (b-c) GTT (n = 3 per group) and ITT (n = 11 per group) of WT and Noc4l^{LOE} mice fed on HFD for 20 or 21 weeks. Statistical analysis was performed with two-way ANOVA with Tukey's test. *p =0.0234 (90 min), *p =0.035 (120 min). All data are presented as mean ± SEM. *p < 0.05, **p < 0.01.

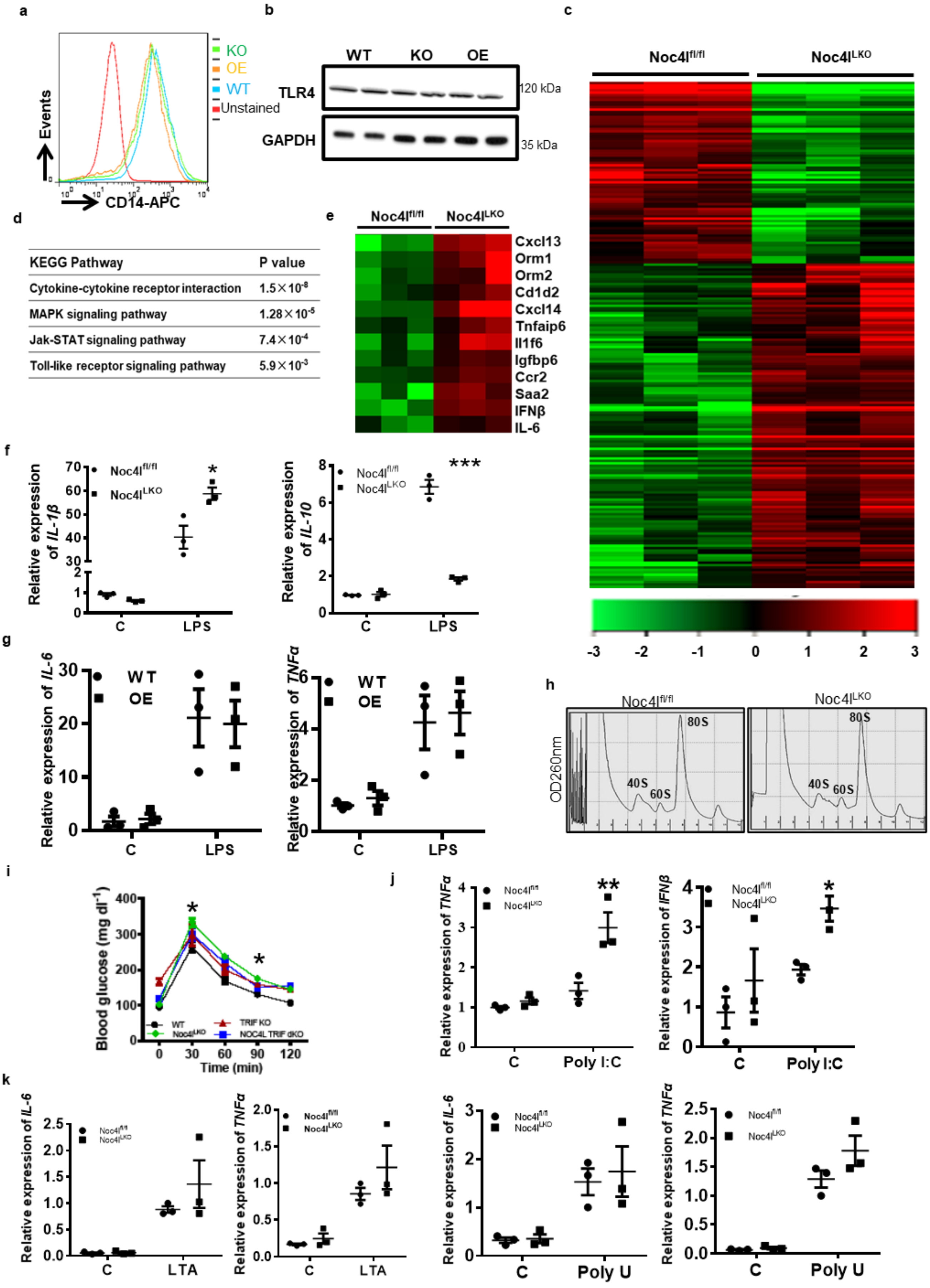
a
KO
OE
WT
Unstained
Events
CD14-APC
b
WT KO OE
TLR4
120 kDa
GAPDH
35 kDa
c
Noc4l^fl/fl
Noc4l^LKO
-3 -2 -1 0 1 2 3
d
KEGG Pathway | P value
Cytokine-cytokine receptor interaction | 1.5×10^-8
MAPK signaling pathway | 1.28×10^-5
Jak-STAT signaling pathway | 7.4×10^-4
Toll-like receptor signaling pathway | 5.9×10^-3
e
Noc4l^fl/fl
Noc4l^LKO
Cxcl13
Orm1
Orm2
Cd1d2
Cxcl14
Tnfaip6
Il1f6
Igfbp6
Ccr2
Saa2
IFNβ
IL-6
f
Relative expression of IL-1β
Relative expression of IL-10
Noc4l^fl/fl
Noc4l^LKO
C
LPS
*
***
g
Relative expression of IL-6
Relative expression of TNFα
WT
OE
C
LPS
h
Noc4l^fl/fl
Noc4l^LKO
OD260nm
40S
60S
80S
i
Blood glucose (mg dl^-1)
Time (min)
WT
Noc4l^LKO
TRIF KO
NOC4L TRIF dKO
*
*
j
Relative expression of TNFα
Relative expression of IFNβ
Noc4l^fl/fl
Noc4l^LKO
C
Poly I:C
**
*
k
Relative expression of IL-6
Relative expression of TNFα
Noc4l^fl/fl
Noc4l^LKO
C
LTA
Poly U

Supplementary Fig. 4 Noc4l was related to inflammation.

(a-b) Flow cytometry of CD14 staining (a) and protein expression of TLR4 (b) of BMDMs from Noc4l$^{fl/fl}$, Noc4l^{LKO} and Noc4l^{LOE} mice (n=3 per group). Gating strategy for flow cytometry was shown in Supplementary Fig. 6b. (c) Microarray analysis of gene expression in unstimulated BMDMs from Noc4l^{LKO} mice, relative to Noc4l$^{fl/fl}$ mice; approximately 200 genes were upregulated upon Noc4l deletion. (d) Pathway analysis of BMDMs from Noc4l^{LKO} vs. Noc4l$^{fl/fl}$ BMDMs according to microarray data. (e) Heatmap of the top 12 genes of differential expression genes of BMDMs from Noc4l$^{fl/fl}$ and Noc4l^{LKO} mice.(f) Relative expression of *IL-1β* and *IL-10* of BMDMs from Noc4l$^{fl/fl}$ and Noc4l^{LKO} mice stimulated with 100 ng/mL LPS for 6 h (n = 3 per group). *p =0.029 (*IL-1β*); ***p =0.0002 (*IL-10*). (g) Relative expression of *IL-6* and *TNFα* in BMDMs from Noc4l$^{fl/fl}$ and Noc4l^{LOE} mice (n = 3 biological replicates). (h) Polysome profiling of BMDMs from Noc4l$^{fl/fl}$ and Noc4l^{LKO} mice. The results are representative of five independent experiments. (i) GTT of Noc4l$^{fl/fl}$, Noc4l^{LKO}, TRIF KO and Noc4l$^{-/-}$/TRIF$^{-/-}$ double KO mice on CD (n=8 per group). Statistical analysis was performed with two-way ANOVA with Tukey's test. *p =0.0102 (30min, Noc4l^{LKO} vs Noc4l$^{-/-}$/TRIF$^{-/-}$ double KO), *p =0.0273 (90 min, Noc4l^{LKO} vs Noc4l$^{-/-}$/TRIF$^{-/-}$ double KO). (j-k) Relative mRNA expression of *TNFα* and *IFNβ* of BMDMs from Noc4l$^{fl/fl}$ and Noc4l^{LKO} mice after treatment with the TLR3 ligand Poly I:C (n = 3 biological replicates) (j), *TNFα* and *IL-6* of BMDM treated with TLR2 ligand LTA, or TLR7 ligand Poly U (n = 3 biological replicates) (k). **p =0.001 (*TNFα)*, *p =0.011 (*IFNβ)* (j). Two-tailed Student's t test (f, g, j, k). All data are presented as mean ± SEM. *p < 0.05, **p < 0.01, ***p < 0.001.

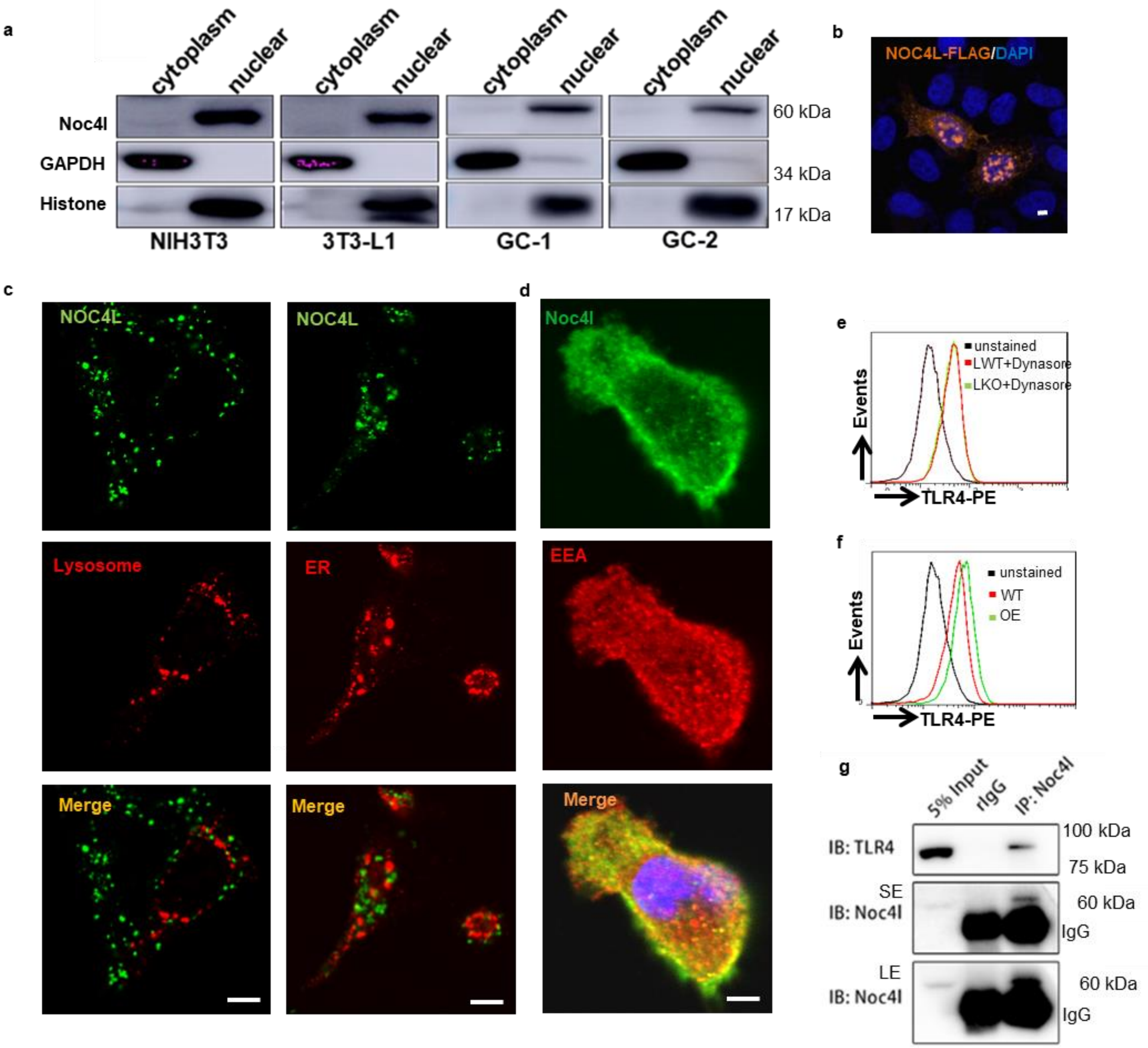
a
cytoplasm
nuclear
Noc4l
GAPDH
Histone
60 kDa
34 kDa
17 kDa
NIH3T3
3T3-L1
GC-1
GC-2
b
NOC4L-FLAG/DAPI
c
NOC4L
Lysosome
ER
Merge
d
Noc4l
EEA
Merge
e
unstained
LWT+Dynasore
LKO+Dynasore
Events
TLR4-PE
f
unstained
WT
OE
Events
TLR4-PE
g
5% Input
rIgG
IP: Noc4l
IB: TLR4
100 kDa
75 kDa
SE
IB: Noc4l
60 kDa
IgG
LE
IB: Noc4l
60 kDa
IgG

Supplementary Fig. 5 The subcellular localization and interaction of NOC4L.

(a) Nuclear and cytosolic extracts were prepared from NIH3T3, 3T3-L1, GC-1 and GC-2 cells and then analyzed for the protein levels of Noc4l, Histone H3 and GAPDH. (b) Immunofluorescence analysis of HeLa cells transfected by NOC4L-flag. (c) Fluorescence microscopy analysis of HeLa cells transfected with GFP-NOC4L plasmid, followed by staining with an ER-Tracker dye (red) and a lysosome-Tracker dye (red). (d) Immunofluorescence staining for Noc4l (3L7, green), EEA (red), and DAPI (blue) in RAW 264.7 cells. (e) Flow cytometry of TLR4 surface staining on PMs from Noc4l$^{fl/fl}$ and Noc4l^{LKO} mice pretreated with dynasore (80μM) for 1 h. (f) Flow cytometry of TLR4 surface staining on PMs from Noc4l$^{fl/fl}$ and Noc4l^{LOE} mice. Gating strategy for flow cytometry was shown in Supplementary Fig. 6b. (g) Endogenous Noc4l and TLR4 interaction in HeLa cells. Cell lysates (Input) and Noc4l (6R) immunoprecipitation analyzed by immunoblot analysis for TLR4 and Noc4l. SE, short exposure and LE, long exposure. Data represented three independent experiments. Scale bar, 5 μm.

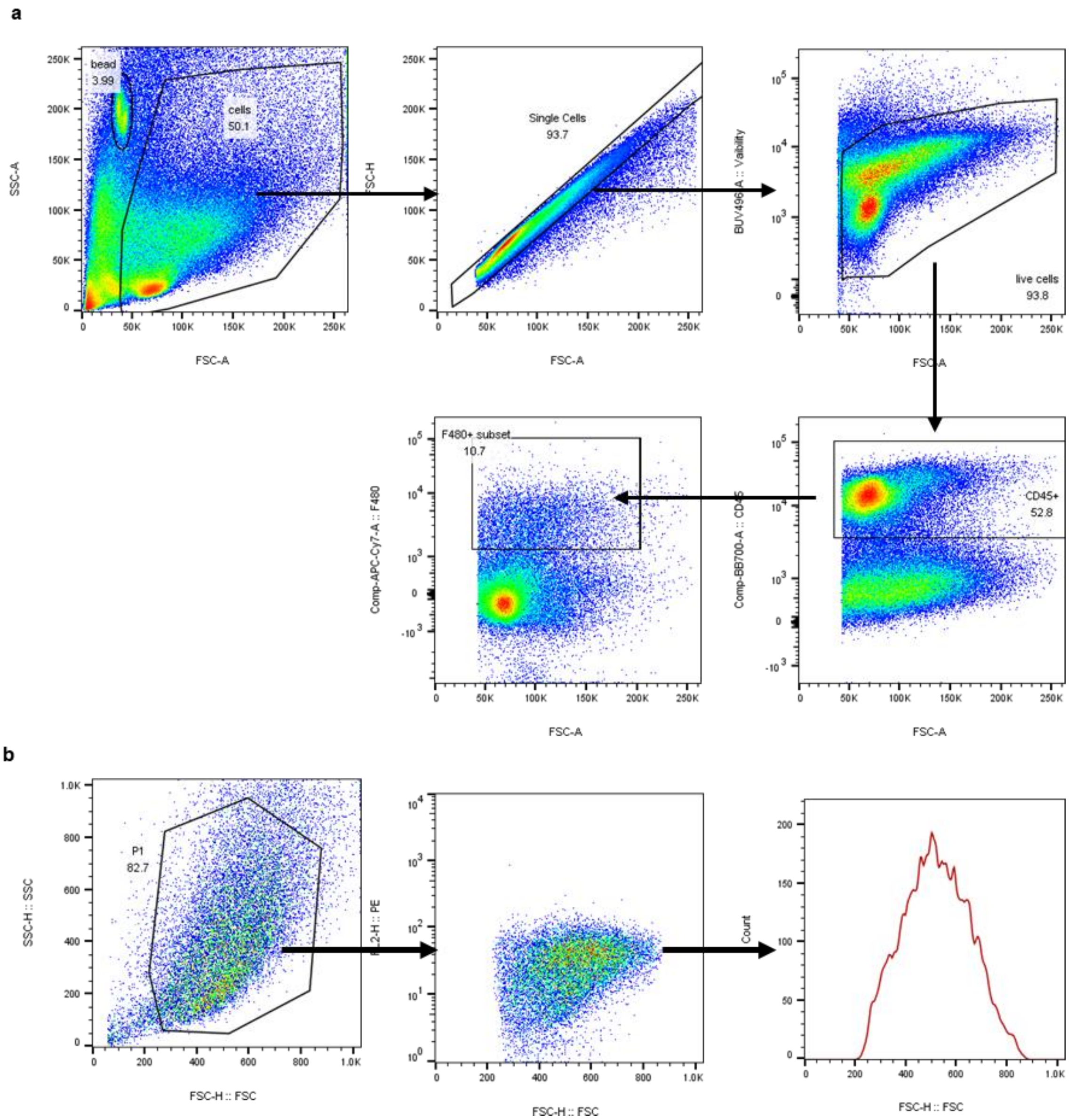

Supplementary Fig. 6 Representative gating strategy for the FACS experiments.

(a) Gating strategy to analyze F4/80$^+$ cells of the SVF isolated from the eWAT of Noc4l$^{fl/fl}$ and Noc4l^{LKO} mice on HFD for 16 weeks presented on Fig. 4e. (b) Gating strategy to analyze TLR4 or CD14 surface staining on PMs presented on Fig. 5n, 5o, Supplementary Fig. 4a and Supplementary Fig. 5e-5f.

Supplementary Table 1

The list of primer sequences for genotyping

Genotype	Name	Sequence of primer (5'- 3')
Noc4l$^{fl/fl}$	loxptF	GCCTTGTCATAGACCATGCGATCTG
	loxptR	TAAGATGCCAGACCGGGGCTTG
Noc4l^{LKO}	loxptF	GCCTTGTCATAGACCATGCGATCTG
	FRTtR	ACAGAACTTGGCTTGAGGCATGTAC
Noc4l^{LOE}	Insulator-tF1	TCCAGGACGGAGTCAGTGAGGCG
	Lyz-promo-tR1	CTCCCAAAATGCTTGGATTAC
TRIF KO	TRIF-F	ACCATGCCCAGATGGTTCAG
	TRIF-R	CTGTGGGAGCCCAAGCTAAG
TLR4	TLR4-F(wild)	ATATGCATGATCAACACCACAG
	TLR4-R(wild)	TTTCCATTGCTGCCCATAG
	TLR4-F(mutant)	GCAAGTTTCTATATGCATTCTC
	TLR4-R(mutant)	CCTCCATTTCCAATAGGTAG

Supplementary Table 2

The list of patient information

	Male	Female	BMI	Age
Lean	√		21.6	22
	√		23.3	54
		√	22.9	35
		√	23.7	52
		√	23.7	50
		√	22.4	26
Obese	√		29.8	29
	√		29.3	32
	√		32.1	57
		√	35.3	35
		√	33.7	40
		√	30.1	72

Supplementary Table 3

The list of primer sequences for real time qRT-PCR

Gene	Sequence of primer (5’- 3’)
mouse GAPDH	F:TGTGTCCGTCGTGGATCTGA
	R:TTGCTGTTGAAGTCGCAGGAG
mouse IL-6	F:TAGTCCTTCCTACCCCAATTTCC
	R:TTGGTCCTTAGCCACTCCTTC
mouse TNFα	F:CTGTAGCCCACGTCGTAGC
	R:TTGAGATCCATGCCGTTG
mouse MCP1	F:AGAGCCAGACGGGAGGAAG
	R:CCAGCCTACTCATTGGGATC
mouse iNOS	F:ACATCGACCCGTCCACAGTAT
	R:CAGAGGGGTAGGCTTGTCTC
mouse Emr1	F:TTGTACGTGCAACTCAGGACT
	R:GATCCCAGAGTGTTGATGCAA
mouse CD68	F:TGTCTGATCTTGCTAGGACCG
	R:GAGAGTAACGGCCTTTTTGTGA
mouse Mgl2	F;AGGCAGCTGCTATTGGTTCTCTGA
	R:AGTTGACCACCACCAGGTGAGAAT
mouse Mrc1	F:TTGGACGGATAGATGGAGGG
	R:CCAGGCAGTTGAGGAGGTTC
mouse Arg1	F:CTCCAAGCCAAAGTCCTTAGAG
	R:GGAGCTGTCATTAGGGACATCA
mouse IFNβ	F:AACCTCACCTACAGGGCGGACTTCA
	R:TCCCACGTCAATCTTTCCTCTTGCTTT
mouse CCL5	F:CTGCCGCGGGTACCATGAAG
	R:TACAGGGTCAGAATCAAG
mouse Noc4l	F:GAGGCAGTGCTGACGAGTC
	R:GAGCCCACGAACAGCTCTTC
human GAPDH	F:ATGGGGAAGGTGAAGGTCG
	R:GGGGTCATTGATGGCAACAATA
human NOC4L	F:GTGGATGAGACACCGCTATCA
	R:GTTGCCTTCCCACTTGGACT

mouse IL-1β	F:GCAACTGTTCCTGAACTCAACT<br>R:ATCTTTTGGGGTCCGTCAACT
mouse IL-10	F:GCTCTTACTGACTGGCATGAG
	R:CGCAGCTCTAGGAGCATGTG
